# Angiotensin-converting enzyme insertion/deletion genotype (rs4646994) association with increased risk of stroke: a case-control study in Eastern Iran

**DOI:** 10.1186/s12883-025-04503-y

**Published:** 2025-11-21

**Authors:** Setayesh MiriMoghaddam, Farzane Vafaeie, Saeede Khosravi Bizhaem, Maryam Yousefi-Roobiyat, Ebrahim Miri-Moghaddam

**Affiliations:** 1 Student Research Committee, Faculty of Pharmacy, Birjand University of Medical Sciences, Birjand, Iran; 2 Student Research Committee, Faculty of Medicine, Cardiovascular Diseases Research Center, Birjand University of Medical Sciences, Birjand, Iran; 3 Cardiovascular Diseases Research Center, Birjand University of Medical Sciences, Birjand, Iran; 4 Department of Neurology, Faculty of Medicine, Birjand University of Medical Sciences, Birjand, Iran; 5 Department of Molecular Medicine, Faculty of Medicine, Cardiovascular Diseases Research Center, Birjand University of Medical Sciences, Birjand, Iran

**Keywords:** Angiotensin converting enzyme, Iran, Ischemic stroke, Polymorphism

## Abstract

**Background and objectives:**

Ischemic stroke (IS), the predominant subtype of stroke, represents a complex and multifactorial disease. While a genetic predisposition is hypothesized to contribute to IS risk, the influence of specific gene variants remains unclear due to inconsistent findings across studies. This study aimed to evaluate the association between the angiotensin-converting enzyme insertion/deletion (*ACE*, I/D) gene polymorphism and the occurrence of IS in a patient cohort from Eastern Iran.

**Methods:**

This case-control study was conducted at the Department of Neurology, Birjand University of Medical Sciences, Eastern Iran. The study population consisted of 406 participants, including 203 individuals diagnosed with IS and 203 age- and gender-matched healthy control subjects. The diagnosis of IS was established based on a comprehensive clinical evaluation, relevant laboratory analyses, neuroimaging, specifically computed tomographyو and magnetic resonance imaging. Genomic DNAs of participants were extracted from peripheral blood samples. The *ACE* I/D genotypes were determined using polymerase chain reaction amplification.

**Results:**

The case group exhibited a higher prevalence of hypertension (71.4%), diabetes mellitus (33.5%), and dyslipidemia (36.9%), compared to the control group. Moreover, the mean systolic blood pressure was significantly elevated in the case group (146 mmHg) in comparison to the control group (123 mmHg). The ID and DD genotypes were more frequent in case subjects (46.6% and 36.8%, respectively) than in control subjects (40.2% and 32.6%, respectively). Conversely, the II genotype was more prevalent in control subjects (27.2%), compared to cases (16.6%). The D allele frequency was significantly higher in the case group (60.1%) than in the control group (52.7%) (*p* = 0.041, 95% CI: 0.554–0.988).

**Discussion:**

This study demonstrated a higher prevalence of the ID and DD genotypes in individuals with IS, compared to control subjects. Furthermore, the D allele frequency was significantly elevated in the IS group.

## Introduction

Cerebrovascular accident (CVA), commonly known as stroke, represents a significant and increasingly prevalent medical condition associated with substantial adult morbidity and mortality. Epidemiological data indicate a marked increase in stroke incidence, with a 50% rise observed over the past 17 years. Current estimates suggest a lifetime risk of approximately one in four individuals developing a CVA [1]. Globally, in a recent assessment year, CVA accounted for a substantial public health burden, with over 13.7 million new cases and 5.5 million deaths. This condition also contributed significantly to global disability, as evidenced by 116.4 million disability-adjusted life years lost [2].

Among the heterogeneous subtypes of CVA, ischemic stroke (IS), characterized by the disruption of cerebral blood flow, constitutes the predominant category, representing approximately 65% of all documented cases [3]. Consequently, the incidence of IS represents a significant global health burden for affected individuals, imposing a substantial emotional and financial burden on families and society. This impact significantly reduces overall well-being across high-, middle-, and low-income countries. In the year 2019, the global prevalence of IS reached 7.63 million individuals, contributing to 3.29 million deaths [4].

The IS pathogenesis, involving the disruption of cerebral blood flow, is secondary to either thrombotic or embolic events. A thrombotic event is characterized by the in situ occlusion of an artery by a thrombus, which can arise from underlying atherosclerotic disease, arterial dissection, fibromuscular dysplasia, or intrinsic inflammatory conditions of the vessel wall. Conversely, an embolic event occurs when embolic material, originating from a distal site within the vasculature, migrates through the bloodstream and subsequently occludes a cerebral vessel, often at a site of pre-existing vascular damage or stenosis [5, 6].

Beyond the direct mechanisms of vascular occlusion, the development of IS is recognized as a multifactorial process influenced by a complex interplay of environmental, physiological, and genetic factors. These include exposure to environmental contaminants, presence of arterial hypertension, inherited genetic susceptibility, and adoption of detrimental lifestyle behaviors. Prior research has demonstrated a significant association between specific genetic determinants and an increased risk of IS [7]. Epidemiological investigations posit that genetic variations contribute to the pathophysiological mechanisms underlying IS, influencing critical pathophysiological alterations [8]. Contemporary research has identified several genetic susceptibility loci associated with an elevated risk of stroke onset, including polymorphisms in genes encoding angiotensin-converting enzyme (*ACE*) [10], alcohol dehydrogenase 1B (*ADH1B*) [11], methylenetetrahydrofolate reductase (*MTHFR*), methionine synthase (*MTR*) [12], interleukin-10 (*IL-10*) [13], and cytochrome P450 family 2 subfamily J member 2 (*CYP2J2*) [14].

The *ACE*, a key component of the renin-angiotensin system, is localized to the endothelium of blood vessels throughout the vasculature and plays a critical role in regulating the proliferation of vascular smooth muscle cells. Its primary enzymatic function is the conversion of angiotensin I to the potent vasoconstrictor angiotensin II. The gene encoding *ACE*, situated at the 17q23.3 locus, spans 26 exons and 25 introns, giving rise to the mRNA transcript that is subsequently translated into the functional *ACE* enzyme [8]. A common genetic variation within the *ACE* gene, designated as the insertion/deletion (I/D) polymorphism (rs4646994), is characterized by the presence (insertion; I allele) or absence (deletion; D allele) of a 287 bp Alu repeat sequence within intron 16 [9]. This I/D polymorphism within the *ACE* gene has the potential to modulate the expression levels of the *ACE* gene transcript and/or influence the enzymatic activity of the resulting angiotensin I-converting enzyme [9].

The genotypic and allelic frequencies of the *ACE* I/D gene polymorphism have demonstrated potential associations with a spectrum of medical conditions, including cardiovascular diseases [10–12], cancer [13, 14], diabetes mellitus [15, 16], and renal diseases [9]. The association between the *ACE* I/D gene polymorphism (rs4646994) and IS susceptibility has not been specifically investigated in the Eastern Iranian population to date. Given the inconsistent findings in the existing literature regarding *ACE* polymorphisms and IS risk, and considering the elevated regional prevalence of IS based on hospital data from this geographical area, the present case-control study aimed to determine the potential association between the *ACE* I/D gene polymorphism and susceptibility to IS in patients from Eastern Iran.

## Materials and methods

### Subjects and study design

This prospective case-control study was conducted from January 2022 to January 2023. The study cohort comprised 203 individuals presenting with an IS. These participants were recruited from the Department of Neurology at Razi Hospital, affiliated with Birjand University of Medical Sciences, located in Birjand, Iran. Ethical approval for this investigation was obtained from the Ethics Committee of Birjand University of Medical Sciences (IR.BUMS.REC.1402.250). Written informed consent was obtained from all participants prior to their inclusion in the study. The study protocol was conducted in accordance with the ethical principles outlined in the Declaration of Helsinki. Inclusion criteria for the case group were age over 18 years and a confirmed diagnosis of acute first-ever or recurrent IS. Exclusion criteria were a diagnosis of hemorrhagic stroke, presence of severe hepatic or renal impairment, known thrombophilia, and refusal to provide informed consent. The diagnosis of IS was established based on a comprehensive evaluation of patient history, clinical presentation, laboratory investigations, and neuroimaging findings obtained through computed tomography and/or magnetic resonance imaging.

Clinical and demographic data–smoking habits, obesity, diabetes, hypertension, hyperlipidemia, cardiac history, myocardial infarction, cardiomyopathy, and neurological conditions, including migraine and transient ischemic attack–were recorded. Stroke severity was assessed using the 15-item National Institutes of Health Stroke Scale (NIHSS), with item scores ranging from 0 (normal) to 4 (completely impaired), although some items were scored from 0 to 2. The NIHSS scores are categorized as follows: 1–5 indicate mild stroke, 6–14 indicate moderate stroke, 15–24 indicate severe stroke, and scores greater than 25 indicate very severe stroke [17]. The control group consisted of 203 normotensive participants, recruited during the study period from individuals attending routine health check-ups. They were matched with the case group for age, gender, residence, and ethnicity.

### Biochemical and genotypes assay

Following an overnight fast, venous blood samples were collected from the antecubital vein into non-anticoagulant tubes for biochemical parameter assays and into tubes containing ethylenediaminetetraacetic acid for DNA extraction. Total cholesterol, high-density lipoprotein (HDL) cholesterol, triglycerides, and plasma glucose concentration were quantified using enzymatic colorimetric assays with commercial reagents, and low-density lipoprotein was calculated using the Friedewald formula. Genomic DNA was isolated using a standard NaCl extraction procedure. The DNA quality and quantity were assessed using a Nano-Drop 2000™ spectrophotometer (Thermo Fisher Scientific, USA). The *ACE* gene I/D polymorphism was analyzed using polymerase chain reaction (PCR). Each 20 µL reaction contained 10 µL of Taq DNA Polymerase 2x Master Mix RED (Ampliqon, Denmark), 0.8 µL of genomic DNA sample, 0.4 µL (10 pmol/µL) of forward primer (5’-CTGGAGACCACTCCCATCCTTTCT-3′), 0.4 µL (10 pmol/µL) of reverse primer (5’-GATGTGGCCATCACATTCGTCAGAT-3′), and 8.4 µL of PCR-grade water. A no-template control, where water replaced DNA, was included in each experiment.

Amplification was performed on an ABI Veriti thermal cycler (Thermo Fisher Scientific, USA) using the following protocol: initial denaturation at 94 °C for 5 min, followed by 30 cycle denaturation at 94 °C for 30 s, annealing at 59 °C for 45 s, and extension at 72 °C for 30 s, with a final extension at 72 °C for 5 min. The PCR products were separated by electrophoresis on 2% agarose gels (SinaClon BioScience, Iran) containing 0.5 mg/L DNA gel stain (SinaClon BioScience, Iran) and visualized using a Uvi-Tec gel documentation system (Biorad, USA). The insertion (I) allele yielded a 490-bp product, and the Deletion (D) allele yielded a 190-bp fragment. Genotypes were identified as follows: II (490-bp band), DD (190-bp band), and ID (490-bp and 190-bp bands).

### Statistical analysis

Data were collected, tabulated, and statistically analyzed using an IBM personal computer and SPSS software version 22 (Armonk, NY: IBM Corp, 2013). Descriptive statistics, including percentage, mean, and standard deviation, were used to present the data. Genotype (DD, ID, II) and allele (D, I) frequencies were calculated for both case and control subjects. Given that the distribution of continuous variables did not meet the assumption of normality, nonparametric statistical tests were employed. Categorical variables were compared using the chi-square test or Fisher exact test, as appropriate. Moreover, the Mann–Whitney U test was utilized for comparisons between two independent groups, while the Kruskal–Wallis test was applied for comparisons across more than two groups. Furthermore, logistic regression analysis was performed to examine the association between independent variables and the dependent outcome. A *p* value of less than 0.05 was considered statistically significant (*p* < 0.05).

## Results 

This case-control study included 203 case (52.7% male, 47.3% female) and 203 control (48.8% male and 51.2% female) subjects. Demographic and clinical characteristics at diagnosis are summarized in Table 1.

**Table 1:** Demographic, clinical, and biochemical characteristics of study participants

Characteristic		Case (*n* = 203)	Control (*n* = 203)	*p* value
Age (years) ^a^		67.6 ± 14.97	65.6 ± 11.31	0.12
Gender ^b^	Male	107 (52.7)	99 (48.80	0.487
	Female	96 (47.3)	104 (51.2)	
BMI (kg/m ^2^) ^a^		25.1 ± 3.45	25.8 ± 5.40	0.353
Hypertension ^b^	Yes	145 (71.4)	0	**< 0.001**
	No	58 (28.6)	203 (100)	
Diabetes ^b^	Yes	68 (33.5)	0	**< 0.001**
	No	135 (66.5)	203 (100)	
Dyslipidemia ^b^	Yes	75 (36.9)	0	**< 0.001**
	No	128 (63.1)	203 (100)	
IHD ^b^	Yes	59 (29.1)	20 (9.9)	**< 0.001**
	No	144 (70.9)	183 (90.1)	
AF ^b^	Yes	17 (8.4)	9 (4.4)	0.105
	No	186 (91.6)	194 (95.6)	
CKD ^b^	Yes	198 (97.5)5 (2.5)	200 (98.5)3 (1.5)	0.724
	No	5 (2.5)198 (97.5)	3 (1.5)200 (98.5)	
SBP (mmHg) ^a^		146.2 ± 27.14	123.7 ± 19.65	**< 0.001**
DBP (mmHg) ^a^		83.9 ± 15.89	76.6 ± 11.28	**< 0.001**
FBS (mg/dl) ^a^		138.57 ± 65.25	97.44 ± 22.62	**< 0.001**
TC (mg/dl) ^a^		156.73 ± 43.21	127.15 ± 32.88	**< 0.001**
TG (mg/dl) ^a^		132.34 ± 72.06	131.08 ± 50.75	0.168
HDL-C (mg/dl) ^a^		43.72 ± 23.09	197.80 ± 36.73	**< 0.001**
LDL-C (mg/dl) ^a^		93.46 ± 35.80	44.21 ± 7.27	**< 0.001**

*BMI* Body mass index, *IHD* Ischemic heart disease, *AF* Atrial fibrillation, *CKD* Chronic kidney disease, *SBP* systolic blood pressure, *DBP* diastolic blood pressure, *FBS* Fasting blood sugar, *TC* total cholesterol, *TG* triglycerides, *HDL-C* high density lipoprotein cholesterol, *LDL-C* low density lipoprotein cholesterol

Categorical variables are expressed as the number (percentage) of subjects and continuous variables are presented as mean ± standard deviation; a: Mann-Whitney was used for continuous variables, b: chi-square (or Fisher exact test) was used for categorical variables. Bolded *p* values are statistically significant (*p* value < 0.05)

Among the 406 participants, DNA samples from 10 cases and 19 control subjects exhibited insufficient quality for genotyping. Consequently, genotyping was successfully performed on the remaining samples. Molecular analysis revealed the ID genotype of the *ACE* gene as the most frequent in both groups, followed by the DD genotype. The II genotype showed a higher prevalence in the control group compared to the case group. The distribution of *ACE* genotypes demonstrated a statistically significant difference between the two groups (Table 2).

**Table 2:** Distribution of angiotensin-converting enzyme genotypes and allele frequencies in the case and control groups

Genotypes and alleles of rs4646994	Control *N* = 184 (%)	Case *N* = 193 (%)	*p* value	95% CI
II	50 (27.2)	32 (16.6)	--	1 (Ref.)
ID	74 (40.2)	90 (46.6)	0.020	0.526 (0.307–0.903)
DD	60 (32.6)	71 (36.8)	0.032	0.541 (0.309–0.948)
Recessive (DI + II)	124 (67.4)	122 (63.2)	--	1 (Ref.)
DD	60 (32.6)	71 (36.8)	0.395	1.203 (0.786–1.840)
Total	184 (100)	193 (100)	--	--
Dominant (DD + DI)	134 (72.8)	161 (83.4)	--	1 (Ref.)
II	50 (27.2)	32 (16.6)	0.013	0.533 (0.323–0.878)
Total	184 (100)	193 (100)	--	--
Heterozygote (DD + II)	110 (59.8)	103 (53.4)	--	1 (Ref.)
DI	74 (40.2)	90 (46.6)	0.210	1.299 (0.863–1.954)
Total	184 (100)	193 (100)	--	--
Allele				
I	174 (47.3)	154 (39.9)	--	1 (Ref.)
D	194 (52.7)	232 (60.1)	0.041	0.740 (0.554–0.988)

Chi-square test and binary logistic regression were applied for categorical comparison of binary outcomes

The association between the *ACE* I/D genotype and clinical and biochemical blood measurements showed that individuals with the DD genotype exhibited higher levels of systolic blood pressure (SBP), total cholesterol, HDL-c, and fasting blood sugar, compared to those with other genotypes. Further details are tabulated in Table 3.

**Table 3:** Association of the *ACE* insertion/deletion genotype with clinical and biochemical characteristics in all participants

Genotypes rs4646994	BMI (kg/m^2^)	SBP (mmHg)	DBP (mmHg)	TC (mg/dl)	TG (mg/dl)	HDL-c (mg/dl)	LDL-c (mg/dl)	FBS (mg/dl)
II	25.9 ± 4.30	131.7 ± 24.85	77.1 ± 15.56	136.5 ± 44.11	133.4 ± 65.04	134.2 ± 81.21	63.9 ± 34.45	113.4 ± 44.24
ID	25.2 ± 4.43	136.1 ± 25.33	80.6 ± 13.06	146.6 ± 40.79	133.1 ± 64.46	114.5 ± 83.18	71.6 ± 35.94	123.2 ± 61.32
DD	25.2 ± 4.46	137.5 ± 28.81	81.3 ± 14.77	141.3 ± 40.61	129.1 ± 62.14	114.2 ± 82.12	71.5 ± 36.90	118.5 ± 50.43
***p*** value (Kruskal-Wallis test	0.282	0.615	0.138	0.117	0.791	0.313	0.068	0.399

*BMI* body mass index, *SBP* systolic blood pressure, *DBP* diastolic blood pressure, *TC* total cholesterol, *TG* triglycerides, *HDL-C* high-density lipoprotein cholesterol, *LDL-C* low-density lipoprotein cholesterol, *FBS* fasting blood sugar, data are presented as mean ± standard deviation

The results showed an association between the *ACE* I/D genotype, under a recessive model, and the presence of hypertension in case groups. Detailed information is presented in Table 4.

**Table 4:** Association of ACE insertion/deletion (I/D) genotype with hypertension under different inheritance models

Genotypes/genetic models	Hypertension		*p* value	95%CI
	No	Yes		
II	8 (14)	24 (17.6)	--	ref
ID	30 (52.6)	60 (44.2)	0.255	1.394 (0.787–2.470)
DD	19 (33.4)	52 (38.2)	0.123	1.591 (0.881–2.871)
Recessive (DI + II/DD)	38 (66.7)	24 (31.6)	< 0.001	0.231 (0.111–0.480)
Dominant (DD + DI/II)	49 (86)	112 (82.4)	0.539	0.762 (0.320–1.814)
Heterozygote (DD + II/DI)	27 (47.4)	76 (55.9)	0.280	1.407 (0.757–2.617)

Chi-square test and binary logistic regression were applied for categorical comparison of binary outcomes

The association of *ACE* I/D genotypes with diabetes mellitus and dyslipidemia under different inheritance models is presented in Tables 5 and 6.

**Table 5:** Association of angiotensin-converting enzyme insertion/deletion (I/D) genotypes with diabetes mellitus under different inheritance models

Genotypes/genetic models/alleles	Diabetes		*p* value	95%CI
	No	Yes		
II	18 (14)	14 (21.8)	--	ref
ID	65 (50.3)	25 (39.1)	0.711	0.874 (0.427–1.787)
DD	46 (35.7)	25 (39.1)	0.712	1.146 (0.557–2.357)
Recessive (DI + II/DD)	83 (64.3)	39 (60.9)	0.644	0.865 (0.466–1.604)
Dominant (DD + DI/II)	111 (86)	50 (78.1)	0.167	0.579 (0.267–1.256)
Heterozygote (DD + II/DI)	64 (49.6)	39 (60.9)	0.137	1.584 (0.861–2.914)

Chi-square test and binary logistic regression were applied for categorical comparison of binary outcomes

**Table 6:** Association of angiotensin-converting enzyme insertion/deletion (I/D) genotype with dyslipidemia under different inheritance models

Genotypes/genetic models/alleles	Dyslipidemia		*p* value	95% CI
	No	Yes		
II	22 (18.3)	10 (13.7)	--	ref
ID	52 (43.4)	38 (52.1)	0.044	2.171 (1.021–4.617)
DD	46 (38.3)	25 (34.2)	0.190	1.698 (0.769–3.749)
Recessive (DI + II/DD)	74 (61.7)	48 (65.8)	0.568	1.194 (0.650–2.191)
Dominant (DD + DI/II)	98 (81.7)	63 (86.3)	0.403	1.414 (0.628–3.185)
Heterozygote (DD + II/DI)	68 (56.7)	35 (47.9)	0.240	0.704 (0.393–1.263)

Chi-square test and binary logistic regression were applied for categorical comparison of binary outcomes

Table 7 presents the association of the *ACE* I/D genotype with stroke subtypes and the degree of arterial stenosis.

**Table 7:** Association of angiotensin-converting enzyme insertion/deletion (I/D) genotype with stroke subtypes and degree of arterial stenosis

Characteristics of stroke		Genotype			*p* value
		II N (%)	DI N (%)	DD N (%)	
Stroke subtype	Unknown etiology	11 (34.4)	45 (50)	37 (52.1)	0.251
	Small artery occlusion (Lacunae)	4 (12.5)	20 (22.2)	15 (21.1)	
	Extracranial atherosclerosis	10 (31.1)	13 (14.4)	7 (9.9)	
	Cardio embolism	4 (12.5)	20 (22.2)	15 (21.1)	
	Intracranial atherosclerosis	3 (9.4)	5 (5.6)	5 (7)	
Degree of arterial stenosis	Normal	13 (40.6)	50 (55.6)	37 (52.1)	0.076
	Plaque without stenosis	5 (15.6)	7 (7.8)	18 (25.4)	
	< 50% stenosis	4 (12.5)	19 (21.1)	9 (12.7)	
	50–70% stenosis	8 (25)	6 (6.7)	3 (4.2)	
	> 70% stenosis	2 (6.3)	8 (8.8)	2 (2.8)	
	Complete occlusion	0	0	2 (2.8)	

Chi-square (Fisher exact) test was used

## Discussion

 The IS is a multifactorial disorder with established risk factors encompassing both modifiable environmental influences and inherent genetic susceptibility. Epidemiological investigations have consistently implicated genetic determinants in modulating the risk of IS. To explore this further, the present case-control study evaluated the *ACE* I/D gene polymorphism in individuals with IS and healthy control subjects within a specific region of Iran. This analysis revealed that the heterozygous ID genotype was the most prevalent in both the case and control cohorts. This observation aligns with those of a prior study conducted by Nouryazdan et al., which reported a similar *ACE* I/D genotype distribution in a Western Iranian population [18]. Furthermore, a meta-analysis encompassing data from 14,000 subjects reported the following frequency ranges for *ACE* I/D genotype: II (22–30%), ID (45–50%), and DD (28–35%) [19]. In the present study, analysis of the data from these populations yielded the following genotype frequencies: DD (27.7%), ID (47.1%), and II (26.2%). These observed frequencies align closely with the ranges reported by Li et al. in their meta-analysis.

This research investigated the association between the *ACE* I/D polymorphism and IS in an Eastern Iranian population. The findings demonstrated a higher prevalence of the ID and DD genotypes among individuals with IS, compared to the control group. Furthermore, the D allele frequency exhibited a statistically significant difference between the case and control groups (*p* < 0.04). In a study performed on an Indonesian population, the DD genotype frequency was reported at 23% in IS cases, compared to 17% in the healthy population [20]. Similarly, Goyal et al.. observed a significant association between the DD genotype and stroke in an Indian population (13.8% vs. 0.8%) [21].

A research conducted by Addisu Melake et al. demonstrated that the *ACE*-DD genotype and the D allele were significantly more common among the patients, compared to the control subjects, with odds ratios of 3.71 (95% CI: 1.02–13.5, *p* < 0.05) and 2.07 (95% CI: 1.06–4.03, *p* < 0.05), respectively [22]. Conversely, Tuncer et al. found no statistically significant differences between IS patients and healthy control subjects in a Turkish population regarding genotype distribution or allele frequency [23]. A meta-analysis of 50 studies (comprising 10,070 case and 22,103 control subjects) conducted by Zhang et al. concluded that the D allele is associated with a modestly increased risk of IS, albeit with low penetrance [24].

Previous research has demonstrated that individuals possessing the II genotype exhibit lower concentrations of the *ACE* enzyme, compared to those with the DD genotype [11]. Individuals carrying the D allele of the *ACE* gene (DD and ID genotypes) exhibited a diminished release of nitric oxide following aerobic exercise, suggesting a potential association among this genetic variation, blood pressure regulation, and endothelial function [25]. Mechanistically, the presence of the D allele is linked to higher *ACE* enzymatic activity and, consequently, increased production of angiotensin II relative to the I allele [25].

Angiotensin II exerts a direct effect on renal sodium homeostasis by augmenting the activity of specific molecules, including the Na+/H + exchanger and Na+/K + ATPase in the proximal tubule, and modulating several ion transporters within the distal nephron and collecting tubules. Furthermore, angiotensin II stimulates the release of aldosterone from the adrenal glands, leading to enhanced reabsorption of sodium and water in renal epithelial cells. This physiological cascade results in an expansion of blood volume and an elevation in blood pressure, potentially contributing to the development of hypertension [26].

Angiotensin II is a potent activator of multiple intracellular signaling pathways, including the mitogen-activated protein kinase cascade, the phosphoinositide 3-kinase/AKT pathway, and cAMP-dependent protein kinase signaling. These pathways play critical roles in the regulation of cell growth, differentiation, cytoskeletal reorganization, and cell cycle progression [27]. Furthermore, increased *ACE* expression in macrophages and smooth muscle cells within coronary artery plaques is recognized as a significant factor in the pathogenesis of ischemic heart disease [18, 28]. This pathophysiological mechanism may similarly elevate the risk of IS in cerebral blood vessels.

Findings of this study indicated a potential association between the DD genotype of the *ACE* gene and elevated blood pressure, defined as SBP >140 mmHg or diastolic blood pressure >90 mmHg, with a prevalence of 38.2% in individuals with hypertension, compared to 33.4% in normotensive individuals. This trend has been reported across multiple populations [29–31]; however, contrasting results have also been observed in some previous studies, which did not identify a similar association [32, 33]. These inconsistencies suggest that factors, such as geographic location, population heterogeneity, ethnicity, and other ecological variables, may influence genotype distribution. Furthermore, environmental factors, including nutrition and physical activity, are known to induce epigenetic modifications. The interplay between these epigenetic alterations and underlying genetic polymorphisms contributes to the complex genetic architecture of blood pressure regulation, potentially explaining the observed variability in association studies concerning the *ACE* I/D gene polymorphism across diverse populations.

This study had certain limitations that warrant consideration. The small sample size may have limited the statistical power to detect subtle associations between the *ACE* genotype and IS. Furthermore, the absence of serum *ACE* activity assays precluded a direct assessment of the functional consequences of the observed genotypes on circulating enzyme levels, potentially impacting the accuracy of the interpretation of the genotype-phenotype relationship. Additionally, the scope of this investigation was restricted by the analysis of a limited number of single-nucleotide polymorphisms. The genetic background and regional factors of the study participants from Eastern Iran may influence allele frequencies and disease associations. Unique ancestry, migration history, and environmental factors such as diet and lifestyle can affect genetic predispositions and disease risk, limiting the generalizability of our findings to other populations. Future studies with multi-regional, large cohorts, diverse genetics, and functional validation— including ACE enzyme activity and mRNA expression—are needed to confirm our results and clarify their broader relevance.

## Conclusion

The results suggest a potential association between the DD genotype of the *ACE* gene and an increased risk of elevated blood pressure. Given the established critical role of *ACE* in the pathogenesis of essential hypertension, a significant risk factor for stroke, the findings indicate that individuals carrying the DD genotype may exhibit heightened susceptibility to developing IS. This observation underscores the importance of considering genetic factors in the development of primary prevention strategies for strokes.

## Data Availability

All data generated or analyzed during this study are included within this published article. Additionally, detailed methodological information is available in the Zenodo repository under the dataset accession number 10.5281/zenodo.17308054.

## Abbreviations

CVA: Cerebrovascular accident
IS: Ischemic stroke
ACE: Angiotensin-converting enzyme
I/D: Insertion/deletion
PCR: Polymerase chain reaction
BMI: Body mass index
IHD: Ischemic heart disease
AF: Atrial fibrillation
CKD: Chronic kidney disease
SBP: Systolic blood pressure
DBP: Diastolic blood pressure
FBS: Fasting blood sugar
TC: Total cholesterol
TG: Triglycerides
HDL-C: High density lipoprotein cholesterol
LDL-C: Low density lipoprotein cholesterol
